# INTERGROWTH-21 Identifies High Prevalence of Low Symphysis–Fundal Height in Indigenous Pregnant Women Experiencing Multiple Infections, Nutrient Deficiencies, and Inflammation: The Maternal Infections, Nutrient Deficiencies, and Inflammation (MINDI) Cohort

**DOI:** 10.1093/cdn/nzab012

**Published:** 2021-04-12

**Authors:** Doris González-Fernández, Elizabeta Nemeth, Emérita del Carmen Pons, Delfina Rueda, Odalis Teresa Sinisterra, Enrique Murillo, Veena Sangkhae, Lisa M Starr, Marilyn E Scott, Kristine G Koski

**Affiliations:** School of Human Nutrition, McGill University (Macdonald Campus), Ste-Anne-de-Bellevue, Quebec, Canada; Center for Iron Disorders, David Geffen School of Medicine, University of California, Los Angeles, CA, USA; Department of Nutritional Health, Ministry of Health, Panama City, Panama; “Comarca Ngäbe-Buglé” Health Region, Ministry of Health, San Félix, Chiriquí Province, Panama; “Panamá Norte” Health Region, Ministry of Health, Panama City, Panama; Department of Biochemistry, University of Panama, Panama City, Panama; Center for Iron Disorders, David Geffen School of Medicine, University of California, Los Angeles, CA, USA; Institute of Parasitology, McGill University (Macdonald Campus), Ste-Anne-de-Bellevue, Quebec, Canada; Institute of Parasitology, McGill University (Macdonald Campus), Ste-Anne-de-Bellevue, Quebec, Canada; School of Human Nutrition, McGill University (Macdonald Campus), Ste-Anne-de-Bellevue, Quebec, Canada

**Keywords:** indigenous pregnant women, symphysis–fundal height, INTERGROWTH-21 standards, inflammation, protein malnutrition, hepcidin

## Abstract

**Background:**

In the absence of ultrasound, symphysis–fundal height (SFH) can assess maternal–fetal well-being as it is associated with gestational age, fetal weight, and amniotic fluid volume. However, other modifiers of SFH, including maternal infections, nutrient deficiencies, and inflammation (MINDI), have not been widely explored.

**Objectives:**

Our objectives were 2-fold: *1*) to assess prevalence of low SFH in indigenous Panamanian women using both Pan-American Health Organization (PAHO) and INTERGROWTH-21 standards and *2*) to explore associations of SFH with maternal health indicators: infections (oral, skin, urogenital, nematode infections), nutrient deficiencies [protein and iron indicators (ferritin, serum iron, serum transferrin receptor, hepcidin), folate, and vitamins A, D, and B-12], and inflammation [leukocytes, C-reactive protein (CRP), cytokines].

**Methods:**

For this cross-sectional study, low-SFH-for-gestational-age was assessed using PAHO and INTERGROWTH <10th centile in 174 women at ≥16 weeks of gestation. Bootstrapping selected MINDI variables for inclusion in multivariable fractional polynomial (MFP) logistic regressions for low SFH. Associations of MINDI variables with hepcidin were also investigated.

**Results:**

Prevalence of low SFH was 8% using PAHO, but using INTERGROWTH, 50.6% had SFH <10th centile, including 37.9% <3rd centile. Both PAHO-SFH <10th centile and INTERGROWTH-SFH <3rd centile were associated with higher hepcidin (OR = 1.12, *P *= 0.008, and OR = 3.04, *P *= 0.001, respectively) and with lower TNF-α (OR = 0.73, *P *= 0.012, and OR = 0.93, *P *= 0.015, respectively). Wood-smoke exposure increased the odds of PAHO-SFH <10th centile (OR = 1.19, *P *= 0.009), whereas higher BMI decreased the odds of INTERGROWTH-SFH <3rd centile (OR = 0.87, *P *= 0.012). Lower pulse pressure (OR = 0.90, *P *= 0.009) and lower inflammatory responses [lower lymphocytes (OR = 0.21, *P *= 0.026), IL-17 (OR = 0.89, *P *= 0.011)] distinguished SFH <3rd centile from SFH ≥3rd to <10th centiles using INTERGROWTH-21 standards. The MFP regression for hepcidin controlling for SFH (adjusted *R*^2^ = 0.40, *P *= 0.001) revealed associations with indicators of inflammation (CRP, *P *< 0.0001; IL-17, *P *= 0.012), acidic urinary pH (*P *= 0.008), and higher intake of supplements (*P *= 0.035).

**Conclusions:**

Associations of low SFH with MINDI variables, including hepcidin, highlight its potential for early detection of multicausal in utero growth faltering.

## Introduction

Clinically measured symphysis–fundal height (SFH) is the first-level screening tool often used for assessing fetal size in marginalized communities in the absence of ultrasound (1). This simple biomarker uses a tape measure in complete contact with the maternal abdomen skin midline to measure the distance between the symphysis pubis and the upper height of the uterine fundus (2). SFH has been strongly correlated with fetal weight and with amniotic fluid volume (3). Public health interest has focused on using SFH as an indicator of small-for-gestational-age (SGA), defined as birth weight <10th centile (4), although a limitation of these previous studies has been the small number of fetuses with growth <10th centile (5).

Presently, SFH is part of the routine pregnancy assessment according to the National Guidelines from the United Kingdom, the United States, Canada, and Ireland (6). In Latin America, the Pan American Health Organization (PAHO) includes SFH standards as part of the recommended routine antenatal care (7). These PAHO guidelines, developed in 1984 by the Latin American Center of Perinatology/Women and Reproductive Health (CLAP/WR) in Montevideo (Uruguay), were based on 1074 SFH measurements during the follow-up of 47 normal pregnant women (white race, medium socioeconomic class, and good nutritional state) (8); they reported a sensitivity of SFH of 52% and a specificity of 92% in the detection of intrauterine growth restriction (7).

Current WHO recommendations state that a normal SFH “may be a reasonable indicator of a healthy baby” (9). However, one of the main concerns for the use of SFH had been the lack of international standardization (10, 11). To address this, the INTERGROWTH-21 project targeted the development of new international SFH standards using a large cohort of 4607 healthy pregnant women from Brazil, China, India, Italy, Kenya, Oman, the United Kingdom, and the United States (12), but comparative studies applying these new standards are scarce.

To date, SFH has been used in low- and middle-income countries for estimating gestational age (GA), guiding clinical decisions in pregnant women treated for *Plasmodium falciparum* malaria (13), and in HIV-infected women for timing the initiation of antiretroviral treatment (14, 15). Lower SFH has been associated with HIV-positive women whose fetuses were infected with HIV (16) and with lower SFH in mothers with severe anemia compared with nonanemic women (17). SFH also has been used for the detection of multiple gestations (18) and for identifying low (oligohydramnios) or high (polyhydramnios) amniotic fluid volume (3). Recently, the intake of iron supplements, higher hemoglobin, and higher diastolic blood pressure were associated with higher SFH *z* scores calculated using INTERGROWTH standards in a Colombian population of mixed ethnic backgrounds (19). However, associations of SFH with other clinical indicators of maternal health have not been investigated.

In Panama, both maternal and infant mortality are highest in indigenous communities, where 36% of infant mortality is due to perinatal causes (20). At the time of this study in 2010, maternal mortality rates in the Ngäbe-Buglé comarca were as high as 200 per 1000 live births (21) and the prevalence of low birth weight in the country was 8.8% (22) but suspected of being higher in the Ngäbe-Buglé indigenous community as 56% of mothers did not have professional delivery attendants (22). We had previously reported in pregnant Ngäbe-Buglé women a high prevalence of maternal infections (23), inflammation, and multiple nutrient deficiencies (24) and an association of low SFH with low maternal pulse pressure (25).

For this cross-sectional study, we applied PAHO's CLAP/WR and the more recent international INTERGROWTH-21 standards to identify the prevalence of low SFH in the Ngäbe-Buglé community. We also explored if co-existing maternal infections, nutrient deficiencies, and inflammation (MINDI) were associated with low SFH using both PAHO-CLAP/WR and INTERGROWTH-21 standards (henceforth, CLAP and INTERGROWTH).

## Methods

### Design

For this cross-sectional study, we recruited pregnant women from the Ngäbe-Buglé indigenous community in the province of Chiriquí Panama from August to December 2010 during their routine pregnancy follow-up. We used 2 approaches to identify low SFH. First, as recommended by the Panamanian Ministry of Health at the time of the study (26), we compared our population with CLAP standards (7) and classified women into 3 groups: SFH <10th, ≥10th to ≤90th centiles (SFH 10–90), and >90th centile for GA. Second, INTERGROWTH allowed us to classify women into 4 groups: <3rd centile, ≥3rd to <10th centiles (SFH 3–10), SFH 10–90, and >90th centile for GA. Comparisons of MINDI variables that differed among these SFH classifications were identified as well as MINDI variables associated with *1*) increasing the odds of SFH <10th and SFH >90th centile [compared with normal SFH (SFH 10–90) using CLAP standards] and with *2*) increasing the odds of SFH <3rd, SFH 3–10, or SFH >90th centile compared with SFH 10–90 using INTERGROWTH standards. MINDI variables specifically associated with the odds of SFH <3rd centile compared with SFH 3–10 were also identified.

### Ethics

Ethics approval was obtained from the Gorgas Memorial Institute in Panama (no. 1618/CNBI/ICGES/10), which required as a prerequisite for final approval a signed agreement with indigenous authorities of the Ngäbe-Buglé comarca and with the Panamanian Ministry of Health. Ethics approval was also obtained from McGill University in Canada (no. A03-M16-10A). All were in accordance with the recommendations of the Operational Guides of Bioethics in Research. Participants gave written informed consent in accordance with the Declaration of Helsinki (27).

### Recruitment

Nurses working at 14 health centers of the Ngäbe-Buglé informed the community of the visit of the research team during the scheduled “pregnancy follow-up day” as most women lived within 2 h walking distance from community health centers. All pregnant women attending these routine check-ups were invited to participate. As an incentive for health promotion from the Panamanian Ministry of Health, all indigenous pregnant women attending prenatal follow-up received food vouchers, and through participation in the project, they were provided with an additional laboratory evaluation without needing to travel to the regional hospital, at a distance of 1–3 h by car. Based on the annual estimate of 2127 live births in the Ngäbe-Buglé community in 2010 (28), we recruited >90% of the pregnant women in the region at the time of the study. Although we had determined that a sample of 92 pregnant women was required to detect an SGA birth with a level of confidence of 95% based on a prevalence of 6.7% for low birth weight reported by the Panamanian Ministry of Health (28), we were able to recruit 213 pregnant women, where 174 were ≥16 weeks’ GA based on last menstrual period, the minimal GA required for assessing SFH using international INTERGROWTH standards (12). Of note, malaria was not endemic and, in all participants, HIV was ruled out by the Ministry of Health and gestational diabetes was ruled out by the absence of glucosuria.

### Questionnaire

Participants answered questions about date of last menstrual period and parity, time in months, and dosage of iron supplementation (60-mg tablets) or a standard multiple nutrient supplement [MNS; tablespoons (tbsp)/d] containing the following in every 100 g (6 tbsp): energy (400 kcal), protein (12.0 g), lipids (12–14 g), vitamin A (250 μg), vitamin E (10 mg), vitamin B-1 (0.50 mg), vitamin B-2 (0.50 mg), vitamin B-3 (6.0 mg), vitamin B-6 (0.50 mg), vitamin B-12 (0.90 μg), folic acid (85 μg), iron (4.0 mg iron bisglycinate), zinc (4.5 mg amino chelated), calcium (100 mg), phosphorus (55 mg), and copper (400 μg). Mothers also reported wood-smoke and fieldwork exposure (hours per day), as well as the number of portions of animal-source foods, leafy vegetables, yellow/red fruits, and vegetables per week, in order to evaluate possible low intake of the nutrients (protein, iron, vitamin B-12, folic acid, and vitamin A) that might be associated with the high prevalence of anemia and malnutrition in the population (29).

### Physical examination

#### Maternal anthropometry

Prepregnancy weights were not available; therefore, BMI at interview was calculated as maternal weight/height squared. Current maternal weight that included the maternal–fetal unit was also used to classify women as underweight, normal weight, or overweight by comparing it with the reference weight for a determined height and GA according to CLAP (26, 30) and used as the standard according to PAHO guidelines (31).

#### Symphysis–fundal height

SFH was measured using a nonelastic tape from the pubic symphysis to the highest point of the uterine fundus while the mother was in a supine position after emptying her bladder. Initially, the tape was placed at the upper border of the symphysis pubis straightened over the uterus until reaching the fundus; measurements were recorded to the nearest complete half centimeter. GA was calculated using the days of amenorrhea according to last menstrual period. The Panamanian guidelines for pregnancy follow-up provide a table with percentiles 10 and 90 of SFH for GA from the 13th to 40th week (26) based on CLAP (7), and this was used to classify women as <10th, ≥10th to ≤90th, and >90th centiles. Also, the SFH calculators developed by the INTERGROWTH-21 Network were used to calculate centiles of SFH for GA expressed in completed weeks of gestation using data ≥16 weeks of gestation and excluding SFH values <12 cm or >38 cm, given that SFH is not clinically accepted beyond these limits (12). Continuous INTERGROWTH centiles for GA were further classified into 4 categories: <3rd, 3–10, 10–90, and >90th centiles. Data from women with GA ≥16 wk were used for analyses for both CLAP and INTERGROWTH standards.

#### Blood pressure and plasma volume calculations

Maternal blood pressure (Omron HEM-790IT automatic BP monitor; IntelliSense®) was measured in a sitting position and considered high if systolic (SBP) ≥140 mm Hg or diastolic (DBP) ≥90 mm Hg and low if SBP <100 and DBP <60 mm Hg (32). Mean arterial pressure (MAP) was calculated as DBP + 1/3 (SBP − DBP) (33) and MAP was considered elevated if >87 mm Hg (10–18 wk), >84 mm Hg (18–34 wk), and >86 mm Hg (>34 wk) (34). Pulse pressure was calculated as the difference between SBP and DBP (35).

For the calculation of plasma volume, we first used Nadler's equation to calculate the total blood volume [TBV = 0.3561 × (height in meters)^3^ + 0.03308 × weight in kilograms + 0.1833] (36). Plasma volume was calculated as TBV × (1 − hematocrit) (37). A low plasma volume was considered if <2 L in the first trimester, <2.6 L in the second trimester, and <2.8 L in the third trimester, according to values less than the fifth centile reported by de Haas et al. (38).

### Infections

Maternal infections were evaluated both clinically and using laboratory measurements as previously described (23). Briefly, caries and skin lesions compatible with scabies were detected during the clinical exam (yes/no). Urinary infection was assessed and scored using microscopic analysis of centrifuged urine. Urine sediment was used for assessing semiquantitative scores (0–4) for urinary bacteria and vaginal Gram stains for semiquantitative scores of *Lactobacillus*, *Bacteroides/Gardnerella*, and *Mobiluncus* to diagnose bacterial vaginosis (Nugent score ≥7) (39). Similarly, scores for vaginal trichomoniasis and diplococcal infection were assessed. Vaginal yeast was detected and scored by direct vaginal smears. The presence of intestinal nematodes (*Ascaris*, hookworm, and *Trichuris*) was identified in the 101 women who provided stool samples using direct microscopic fecal examination and Kato-Katz and Flotac tests as previously described (23).

### Laboratory analyses

For the evaluation of hematological status and inflammation, complete RBC and white blood cell (WBC) counts (BC-5500 Mindray Auto Hematology Analyzer; Mindray (UK) Ltd) were performed. Anemia was defined as hemoglobin <110 g/L (40). Eosinophilia, a proxy for intestinal nematode infection, was defined as eosinophil count >0.6 × 10^3^/mm^3^ (41).

Serum was analyzed for C-reactive protein (CRP) using solid-phase ELISA (MP Biomedicals) with a minimum detectable concentration of 0.95 nmol/L and for cytokines IL-1β, IL-4, IL-6, IL-10, IL-12, IL-13, IL-17, IFN-γ, TNF-α, and monocyte chemotactic protein 1 (MCP-1) that were quantified via Luminex using a Human Cytokine/Chemokine Magnetic Bead Panel (Millipore Corporation Canada).

Maternal iron status was evaluated based on serum iron (spectrophotometry, FERENE-ENDPOINT, IRON-SL kit; Sekisui Diagnostics), ferritin (ELISA; MP Biomedicals), and serum transferrin receptor (sTfR; RAMCO immunoassay; RAMCO Laboratories). Other serum nutrients evaluated were as follows: *1*) folic acid and vitamin B-12 concentrations using immunoelectro-chemiluminescence (MODULAR E170; Roche Diagnostics); *2*) 25-hydroxyvitamin D using the LIAISON®, DiaSorin, direct competitive chemiluminescence immunoassay; *3*) vitamin A using HPLC (42); and *4*) retinol-binding protein (RBP) using Human RBP4 ELISA (MP Biomedical) with a standard curve range between 0.14 and 100 ng/mL. Hepcidin was measured using an Intrinsic Hepcidin IDx ELISA kit (Intrinsic LifeSciences).

Cutoffs for iron status indicators were set as serum iron <8.9 µmol/L, ferritin <20 µg/L (40), or elevated sTfR (>8.3 mg/L) (RAMCO Laboratories, INC). Folic acid deficiency was defined as <10 nmol/L and vitamin B-12 deficiency as <150 pmol/L (43). We used a cutoff for vitamin D deficiency of <50 nmol/L (44). The cutoff for low vitamin A was set at <1.05 µmol/L (45) and low protein status was defined as RBP <30 mg/L (46), which is considered a sensitive indicator of protein status even before clinical signs of malnutrition appear (47).

Urine samples were assessed using URISCAN strips (YD Diagnostics) on a Miditron-M semiautomated reflectance photometer (Roche) for semiquantitative measurement of leukocyte esterase (positive reaction considered as urinary tract infection) (48), urinary-specific gravity (considered hypohydration if >1.020) (49), and urinary pH as a general indicator of the acid–base balance (50) and of volemic status given its positive correlation with extracellular volume (51).

### Statistical analysis

All statistical analyses were performed using STATA 16 (StataCorp). Differences in maternal characteristics and nutritional, inflammation, and infection status were assessed among women having SFH <10th, ≥10th to ≤90th, and >90th centiles (CLAP) and SFH <3rd, SFH 3–10, SFH 10–90, and >90th centiles (INTERGROWTH). One-factor ANOVA or Kruskal-Wallis analyses were used depending on the nature of continuous variables. Frequency comparisons of discrete variables were made among classifications using chi-square or Fisher's exact tests. We report *P* values at <0.05 and at the Bonferroni-corrected *P* value calculated for each group of these analyses. We also compared the continuous variable for SFH centiles for GA, inflammation, and iron status indicators by the presence/absence of protein deficiency, indicated by RBP <30 mg/L, using Kruskal-Wallis tests.

### Selection of independent variables and modeling strategy

We began by grouping independent variables as follows: *1*) maternal characteristics (GA, maternal age, parity, BMI, exposure to wood smoke and fieldwork), *2*) supplementation and diet (months on iron supplements, MNS tbsp/d, weekly portions of animal-source foods, and yellow/red and green-leafy fruits and vegetables), *3*) blood cell counts and differentials (WBCs, RBCs, and platelets), *4*) cytokines (IL-1β, IL-4, IL-6, IL-10, IL-12, IL-13, IL-17, TNF-α, INF-γ, MCP-1), *5*) serum nutritional indicators (RBP, folic acid, and vitamins A, B-12, and D), *6*) iron status indicators (ferritin, sTfR, serum iron, hepcidin), *7*) vaginal micro-organisms (*Lactobacillus, Bacteroides/Gardnerella, Mobiluncus, Trichomonas*, yeast, *Diplococcus*), *8*) urinary indicators (urinary pH, specific gravity, bacteria, leukocytes, crystals), and *9*) other infections (caries, scabies). The subsample of women with data on intestinal nematodes (*Ascaris*, hookworm, *Trichuris*) was tested separately.

We selected variables from each of the above-described groups using a bootstrap procedure. The nonparametric bootstrap procedure resamples observations with replacements, drawing repeated bootstrap samples from the original dataset (52). We applied a backward stepwise algorithm to count the total number of times variables from each group were selected after 1000 repetitions, and preliminary candidate variables entering ≥500 times (50%) (53) were used to run multivariate fractional polynomial (MFP) logistic regression models.

### MFP modeling approach

Preparative analyses had shown nonlinear associations between our biological measurements and outcome variables. To address the issue of nonlinearity, we used an MFP algorithm for assessing associations of our MINDI variables with low and high SFH. The MFP compares functional fractional polynomial transformations of predictor variables and selects the most robust ones by determination of suitable forms of continuous independent variables while correcting nonlinear associations (54). The final MFP backwards selection of variables was set at *P *< 0.25 to avoid missing variables of possible importance (55). SFH centile variables already adjust for GA, but given the variation in independent variables during pregnancy and the variation in the interpretation of SFH for the detection of SGA depending on GA (56), models were controlled by trimester. Postestimation graphs describing the odds of low SFH with significantly associated nutritional and inflammation biomarkers were drawn. We followed a similar procedure to run an MFP linear regression model for log-hepcidin as a dependent variable while controlling for GA in weeks and for SFH in centimeters. For all models, absence of collinearity and instability of regression coefficients were evaluated using a variance inflation factor (VIF) <10 and a condition number <30, respectively. Little's chi-square test was used when intestinal parasites were included to evaluate randomness of missing data (57).

## Results

### Population characteristics

Maternal characteristics are described in **Table 1**. Women had a median length of gestation of 30 wk (range: 16.3–42 wk) and 37.4% were in their second and 62.6% were in their third trimester. The majority (63.8%) had normal weight, 24.1% had high weight, and 12.1% had low weight for GA by PAHO standards. Adolescent pregnancies (≤19 y, 26.4%) and multiparity (≥5 pregnancies, 33.3%) were present. Most women (92%) were exposed to wood smoke used for cooking (median = 2 h/d; range: 0–6 h/d) and 51.6% did fieldwork (median: 1 h/d; range: 0–10 h/d). Although hypertension using SBP and DBP was not found, hypotension (blood pressure <100/60 mm Hg) occurred in 24% and elevated MAP by GA in 12.6%. Plasma volume was low for GA in 99.4% of pregnancies, and urinary-specific gravity >1.020, indicating hypohydration, occurred in 25.4%.

**FIGURE 1:**
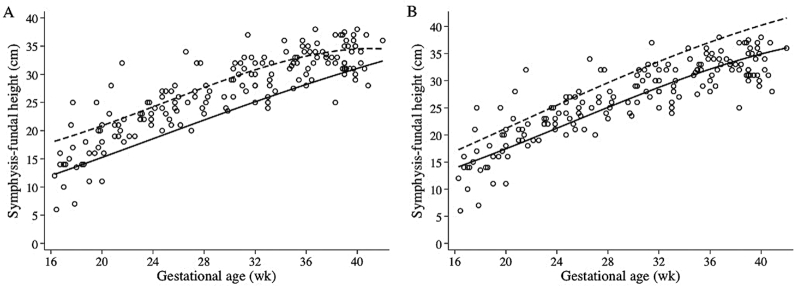
Scatterplot of symphysis–fundal height for gestational age. Solid and dashed lines correspond to the 10th and 90th centiles, respectively, for gestational age according to the CLAP/WR (A) and INTERGROWTH-21 (B) standards. CLAP/WR, Latin American Center for Perinatology/Women and Reproductive Health.

**FIGURE 2:**
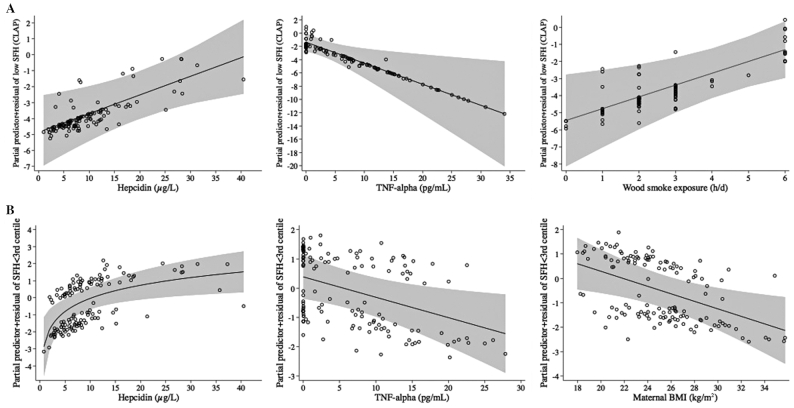
Fractional polynomial logistic regression lines with 95% CIs (gray areas) for asssociations of hepcidin, TNF-α, and wood smoke in the model with SFH <10th centile according to CLAP (A) and the associations of hepcidin, TNF-α, and BMI with SFH <3rd centile according to INTERGROWTH (B), compared with respective SFH between the 10th and 90th centiles, adjusted for all covariates in respective models. CLAP, Latin American Center of Perinatology; SFH, symphysis–fundal height.

**TABLE 1:** Maternal characteristics of indigenous Ngäbe-Buglé pregnant women^1^

	Values
General characteristics	
Age, y	24 (13–45)
Parity, *n*	3 (1–12)
Gestational age, wk	30.8 (16.3–42)
Unadjusted BMI, kg/m^2^	24.8 (17.1–35.8)
Underweight, %	12.1
Normal weight, %	63.8
Overweight, %	24.1
Fieldwork, h/d	1 (0–10)
Wood-smoke exposure, h/d	2 (0–6)
Foods, <7 servings/wk, %	
Animal-source foods	81.0
Yellow/red fruits and vegetables	91.5
Green-leafy vegetables	89.1
Supplementation	
Taking iron supplements, %	85.6
Months on iron supplements	2 (0–10)
Taking MNS supplements, %	56.3
MNS, tbsp/d	1 (0–9)
Infections, %	
Caries	19.5
Scabies	19.0
UTI (*n *= 169)	63.9
*Ascaris* (*n *= 101)	32.7
Hookworm (*n *= 101)	59.4
*Trichuris* (*n* = 101)	13.9
Vaginal microflora (*n* = 173), %	
* Lactobacillus*	53.8
*Bacteroides/Gardnerella*	93.1
*Mobiluncus*	84.4
*Trichomonas*	76.3
Yeast	23.6
*Diplococcus*	21.4
Inflammation indicators	
CRP, mg/L	3.6 (1.5–6.7)
Elevated CRP,^2^ %	12.1
Cytokines, median (IQR), pg/mL	
IL-1β	1.8 (0.5–8.4)
IL-4	5.0 (2.3–19.2)
IL-6	1.6 (1.6–13.1)
IL-10	1.6 (0.1–4.8)
IL-12	1.8 (0.03–25.9)
IL-13	1.6 (0.1–7.4)
IL-17	2.2 (0.1–10.8)
TNF-α	6.7 (0.02–12.4)
IFN-γ	3.6 (1.0–13.4)
MCP-1	175 (115–274)
Blood pressure, median (IQR), mm Hg	
Systolic	102 (96–110)
Diastolic	60 (56–68)
Low blood pressure,^3^ %	24.1
MAP, median (IQR)	74.3 (70.0–81.3)
Elevated MAP,^4^ %	12.6
Pulse pressure	40 (35–47)
Other perfusion indicators	
Plasma volume, mL	2106 (1733–2621)
Low plasma volume,^5^ %	99.4
Urinary-specific gravity	1.020 (1.005–1.030)
Urinary-specific gravity >1.020, %	25.4
Urinary pH	6 (5–8)
Nutritional status indicators^6^	
Ferritin, µg/L	9.9 (5.5–20.4)
Ferritin <20 µg/L, %	73.6
sTfR, mg/L	5.3 (3.9–7.3)
sTfR >8.3 mg/L, %	18.4
Serum iron, µmol/L	8.4 (5.4–13.6)
Serum iron <8.9 µmol/L, %	53.4
Folic acid, nmol/L	13.3 (9.8–18.2)
Folic acid <10 nmol/L, %	26.4
Vitamin B-12, pmol/L	95.5 (79.0–120.0)
Vitamin B-12 <150 pmol/L, %	89.1
Vitamin D, nmol/L	45.1 (33.5–56.2)
Vitamin D <50 nmol/L, %	62.6
Vitamin A, µmol/L	1.1 (0.9–1.4)
Vitamin A <1.05 µmol/L, %	41.9
RBP	44.1 (28.3–84.0)
RBP <30 mg/L, %	28.9
Hepcidin, µg/L	7.4 (5.0–11.3)
Blood cell counts^6^	
RBCs, ×10^6^/mm^3^	3.71 (3.5–3.9)
Hemoglobin, g/L	112 (104.0–118.0)
Anemia, %	37.9
Hematocrit, %	34.9 (33.1–36.6)
MCV, fL	94.6 (91.2–98.0)
MCH, pg	30.3 (29.1–31.4)
MCHC, g/L	319.0 (311.0–326.0)
RDW-CV, %	13.4 (13.1–13.7)
Platelets, ×10^3^/mm^3^	255.5 (73–445)
Total WBCs, ×10^3^/mm^3^	8.5 (7.2–9.8)
Neutrophils	5.9 (4.6–6.8)
Lymphocytes	1.9 (1.7–2.3)
Monocytes	0.4 (0.3–0.4)
Eosinophils	0.3 (0.2–0.5)
Eosinophils >0.6, %	14.9
Basophils	0.03 (0.02–0.04)

1 Values are medians (range) unless otherwise indicated. CRP, C-reactive protein; MAP, mean arterial pressure; MCH, mean corpuscular hemoglobin; MCHC, mean corpuscular hemoglobin concentration; MCP-1, monocyte chemotactic protein 1; MCV, mean corpuscular volume; MNS multiple nutrient supplement; RBP, retinol-binding protein; RDW-CV, red blood cell distribution width–CV; sTfR, serum transferrin receptor; tbsp, tablespoons; UTI, urinary tract infection (detected by positive urinary leukocyte esterase); WBC, white blood cell.

2 Elevated CRP: >3 mg/L in the first, >20.3 mg/L in the second, and >8.1 mg/L in the third trimester (41).

3 Low blood pressure: <100/60 mm Hg (35).

4 Elevated MAP: >87 mm Hg (10–18 wk), >84 mm Hg (18–34 wk), and >86 mm Hg (>34 wk) (34).

5 Low plasma volume: <2 L in the first, <2.6 L in the second, and <2.8 L in the third trimester (38).

6 Values are medians (IQR) unless otherwise noted.

Table 1 also shows the presence of multiple infections, nutrient deficiencies, and inflammation in our MINDI cohort. Infections were highly prevalent; 96.0% had ≥2 co-occurring infections, including bacterial, protozoal, fungal, or nematode infections. Although, in general, WBC indices were within normal limits, we found eosinophilia, which is commonly associated with nematode infections, in 14.9% and elevated CRP in 12.1%.

Diet quality was suboptimal. Women had low weekly intakes of animal-source foods (median = 2 portions/wk; range: 0–14 portions/wk), green-leafy vegetables (median = 1 portion/wk; range: 0–8 portions/wk), and yellow/red fruits and vegetables (median = 1 portion/wk; range: 0–14 portions/wk). Iron tablets (elemental iron, 60 mg) had been taken for a median of 2 mo (range: 0–10 mo), and women consumed 1 tbsp/d of MNS (median: 1; range: 0–9 tbsp/d) out of the recommended 6 tbsp/d. Despite availability of supplements, 38% had anemia, 73.6% had low ferritin, and 53.4% had low serum iron, but only 18.4% had elevated sTfR. Median hepcidin concentration was 7.38 µg/L (range: 0.30–40.49 µg/L). Multiple micronutrient deficiencies [vitamin B-12 (89.1%), vitamin D (62.6%), vitamin A (41.9%), and folic acid (26.4%)] were present.

### Prevalence of low SFH by CLAP and INTERGROWTH standards

Scatterplots of SFH for GA with fitted lines showing the 10th and 90th centiles according to CLAP and INTERGROWTH standards are shown in **Figure 1**A and B, respectively.

The prevalence of SFH <10th centile was 8.0% and that of SFH >90th centile was 28.7% using CLAP. In contrast, when using INTERGROWTH, the prevalence of SFH <3rd centile was 37.9%, 12.6% were classified as SFH 3–10, 40.2% had SFH 10–90, and 9.2% had SFH >90th centile.

### Differences in MINDI variables among SFH classifications

Comparisons among SFH <10th, between the 10th and the 90th, and >90th centiles using CLAP are summarized in **Supplemental Table 1**. Using CLAP, few variables showed differences at *P* < 0.05: mothers with SFH <10th centile had more acidic urine (*P *= 0.015), higher hepcidin (*P *= 0.044), lower TNF-α (*P *= 0.007), lower IL-1β (*P *= 0.014), and lower than expected vitamin D deficiency (*P *= 0.013).Using a Bonferroni-corrected *P *< 0.001, only underweight was found more often than expected in women with SFH <10th centile when compared with SFH ≥10th centile (*P *< 0.0001).

Comparisons among SFH <3rd, 3–10, 10–90, and >90th centiles using INTERGROWTH are shown in **Supplemental Table 2**. More variables differed at *P *< 0.05 across the INTERGROWTH SFH classification: urinary pH was lower in women with SFH <3rd centile (*P *= 0.040) and maternal BMI was lower in women with SFH <3rd centile (*P *= 0.006). We also observed a trend of women with SFH <3rd centile to have lower pulse pressure (*P *= 0.052). Despite a similar prevalence of individual infections, WBC total count, and differential and CRP concentrations, some cytokines differed across SFH classifications. Women with SFH 3–10 had higher concentrations of IL-6 (*P *= 0.025) and IL-17 (*P *= 0.024) compared with those with SFH <3rd and SFH 10–90. They also had higher IL-12 (*P *= 0.018) and TNF-α (*P *= 0.010) compared with mothers with SFH <3rd centile. On the other hand, IL-4 was lower in the SFH <3rd centile compared with SFH >90th centile (*P *= 0.045). With regard to nutritional indicators, RBP concentrations were lower in the SFH <3rd centile compared with the >90th centile (*P *= 0.017) and protein deficiency (RBP <30 mg/L) was higher than expected in the SFH <3rd centile (*P *= 0.048); however, vitamin B-12 concentrations were higher in the SFH <3rd centile when compared with SFH 10–90 (*P *= 0.042). Finally, mothers with an SFH >90th centile had an earlier GA compared with those <10th centile and were less frequently exposed to wood smoke. However, using a Bonferroni-corrected *P *< 0.001, no variables differed.

### Protein deficiency (RBP ≤30 mg/L) and biomarkers of inflammation and iron status

Binary comparisons of SFH centiles (continuous variable) and indicators of inflammation and iron status by presence/absence of protein deficiency are shown in **Table 2**. Using the Bonferroni-corrected *P *< 0.002 for significance, protein-deficient women had lower basophil counts (*P *= 0.0003) and higher IL-13 concentrations (*P *= 0.0009) than protein-sufficient women. At *P *< 0.05, the SFH centile for GA was lower in protein-deficient women (*P *= 0.029), who also had a lower eosinophil count (*P *= 0.032) and lower concentrations of IL-4 (*P *= 0.005) but higher IL-10 (*P *= 0.006). CRP, hepcidin, or iron status indicators did not differ by protein status.

**TABLE 2:** Binary comparisons by RBP <30 mg/L (protein deficient) or RBP ≥30 mg/L (protein sufficient) of SFH centiles for GA and inflammation and iron status indicators in 174 indigenous pregnant women^1^

	RBP <30 mg/L	RBP ≥30 mg/L	*P*
SFH centile for GA	3.0 (0.1–30.3)	10.3 (0.9–52.5)	0.029
White blood cells, ×10^3^/mm^3^	8.2 (7.3–9.8)	8.5 (7.0–9.9)	0.621
Neutrophils	5.6 (4.7–6.9)	5.9 (4.6–6.7)	0.907
Lymphocytes	1.9 (1.7–2.2)	1.9 (1.7–2.3)	0.386
Monocytes	0.3 (0.3–0.4)	0.4 (0.3–0.4)	0.648
Eosinophils	0.2 (0.2–0.4)	0.4 (0.2–0.5)	0.032
Basophils	0.02 (0.02–0.03)	0.03 (0.02–0.04)	0.0003
Cytokines, pg/mL			
IL-1β	2.5 (0.1–9.8)	1.6 (0.6– 7.0)	0.936
IL-4	3.2 (0.7–10.7)	9.1 (3.2–22.7)	0.0049
IL-6	1.6 (0.5–9.9)	1.6 (1.6–13.8)	0.307
IL-10	1.6 (1.0–5.9)	1.0 (0.06–3.9)	0.006
IL-12	0.6 (0.02–28.7)	1.9 (0.06–21.0)	0.704
IL-13	1.6 (1.6 –9.0)	1.0 (0.08–7.2)	0.0009
IL-17	4.5 (0.1–13.5)	2.1 (0.1–10.0)	0.321
TNF-α	5.6 (0.02–12.3)	6.7 (0.02–12.7)	0.882
IFN-γ	5.7 (0.9–14.3)	2.2 (1.0–13.4)	0.899
CRP, mg/L	4.3 (2.0–6.7)	3.2 (1.5–6.7)	0.583
Hepcidin, µg/L	7.0 (5.5–12.2)	7.7 (5.0–10.9)	0.949
Ferritin, µg/L	8.7 (5.3–19.3)	11.4 (5.5–23.7)	0.392
Serum iron, µmol/L	8.2 (6.0–13.5)	8.7 (5.4–14.1)	0.989
sTfR, mg/L	6.1 (3.9–7.9)	5.0 (3.9–6.8)	0.176

1 Values are medians (IQR). One-factor ANOVA was used when the variable was normally distributed (total white blood cells, neutrophils, lymphocytes and basophils), and Kruskal-Wallis tests were used for non-normally distributed variables. CRP, C-reactive protein; GA, gestational age; RBP, retinol-binding protein; SFH, symphysis–fundal height; sTfR, serum transferrin receptor.

### MFP logistic regression models for SFH classifications

#### Model for low SFH according to CLAP

As shown in **Table 3**, higher hepcidin concentrations as well as increased hours per day of wood-smoke exposure were associated with increased odds of an SFH <10th centile compared with an SFH between the 10th and 90th centiles. However, higher TNF-α concentrations decreased the odds. Regression lines showing the odds of an SFH <10th centile with higher hepcidin, TNF-α, and wood smoke, controlling for model covariates, are shown in **Figure 2**A.

**TABLE 3:** MFP logistic regression model for SFH <10th centile compared with SFH between the 10th and 90th centiles using CLAP standards^1^

	OR ± SE	*P*	95% CI	Change in odds for SD increase in *X*	Overall model
Trimester (0 = second, 1 = third trimester)	0.72 ± 0.56	0.675	0.16, 3.33	0.85	*P* < 0.0001, pseudo-*R*^2 ^= 0.383
*t* Wood smoke, h/d	1.99 ± 0.52	0.009	1.19, 3.34	2.79	
*t* TNF-α, pg/mL	0.73 ± 0.09	0.012	0.57, 0.93	0.08	
*t* Urinary pH	0.48 ± 0.20	0.087	0.20, 1.11	0.52	
*t* Hepcidin, µg/L	1.12 ± 0.05	0.008	1.03, 1.22	2.27	
Constant	0.02 ± 0.02	<0.0001	0.004, 0.14		

1 Variables that entered ≥500 bootstrap repetitions but were taken out by the MFP process: multiple nutrient supplement (tbsp/d). The MFP process provided the following equations for transforming covariates (*t*):

Wood smoke (h/d) = wood smoke − 2.5; TNF-α (pg/mL) = TNF-α − 7.433294524; urinary pH = urinary pH − 6.423728814; hepcidin (µg/L) = hepcidin − 9.676559332. Model VIF: 1.05; condition number: 2.74, *n* = 118. VIF: variance inflation factor as measurement of collinearity. Values <10 were accepted. Condition numbers <30 were accepted as measurement of stability of coefficients. CLAP, Latin American Center of Perinatology; MFP, multivariable fractional polynomial; SFH, symphysis–fundal height; tbsp, tablespoons.

#### Models for low SFH according to INTERGROWTH

An SFH <3rd centile yielded significant associations with MINDI in 3 different models. First, **Table 4**A shows associations of an SFH <3rd centile compared with SFH 10–90, where 3 main associations of SFH <3rd centile emerged, 2 of them in common with the CLAP SFH <10th centile. Higher hepcidin was associated with increased odds, whereas higher maternal BMI and TNF-α were associated with decreased odds of an SFH <3rd centile. Model associations controlling for covariates are shown in postestimation regression lines (Figure 2B). In a second model for the subsample of women with data on intestinal nematodes (**Supplemental Table 3**), the association between an SFH <3rd centile and hepcidin remained and the presence of *Trichuris* was associated with 5.5 ± 4.8 (*P *= 0.049) increased odds of an SFH <3rd centile.

**TABLE 4:** MFP logistic regression model for (A) SFH <3rd centile compared with SFH 10–90 and (B) SFH <3rd centile compared with SFH 3–10 and (C) SFH 3–10 compared with SFH 10–90, using INTERGROWTH-21 standards^1^

	OR ± SE	*P*	95% CI	Change in odds for SD increase in *X*	Overall model
(A) SFH <3rd centile compared with SFH 10–90^2^					
Trimester (0 = second, 1 = third trimester)	1.62 ± 0.67	0.245	0.72, 3.65	1.26	*P* < 0.0001, pseudo-*R*^2 ^= 0.164
*t* BMI, kg/m^2^	0.87 ± 0.05	0.012	0.78, 0.97	0.58	
*t* TNF-α, pg/mL	0.93 ± 0.03	0.015	0.88, 0.99	0.60	
*t* Hepcidin, µg/L	3.04 ± 1.03	0.001	1.56, 5.91	2.11	
Constant	0.91 ± 0.30	0.773	0.48, 1.72		
(B) SFH <3rd centile compared with SFH 3–10^3^					
Trimester (0 = second, 1 = third trimester)	106 ± 0.80	0.936	0.24, 4.62	1.03	*P* < 0.0001, pseudo-*R*^2 ^= 0.315
*t* Pulse pressure, mm Hg	0.90 ± 0.03	0.009	0.83, 0.97	0.41	
*t* Lymphocyte count, ×10^3^/mm^3^	0.21 ± 0.15	0.026	0.05, 0.83	0.49	
*t* IL-17, pg/mL	0.89 ± 0.04	0.011	0.82, 0.97	0.39	
*t* MCP-1, pg/mL	0.99 ± 0.003	0.005	0.98, 0.99	0.39	
Constant	5.20 ± 3.27	0.009	1.52, 17.83		
(C) SFH 3–10 compared with SFH 10–90^4^					
Trimester (0 = second, 1 = third trimester)	2.63 ± 1.81	0.158	0.69, 10.12	1.60	*P* = 0.0018, pseudo-*R*^2 ^= 0.170
*t* Age, y	0.90 ± 0.04	0.034	0.81, 0.99	0.46	
*t* Fieldwork, h/d	0.77 ± 0.12	0.088	0.57, 1.04	0.49	
*t* Ferritin, mg/L	1.09 ± 0.01	0.006	1.01, 1.07	2.43	
*t* IL-12, pg/mL	1.04 ± 0.01	0.008	1.01, 1.07	2.28	
Constant	0.09 ± 0.06	<0.0001	0.02, 0.32		

1 MCP-1, monocyte chemotactic protein 1; MFP, multivariable fractional polynomial; SFH, symphysis–fundal height; VIF, variance inflation factor.

2 Model A: Variables that entered ≥500 bootstrap repetitions but were taken out by the MFP process: maternal systolic blood pressure (mm Hg). The MFP process provided the following equations for transforming covariates (*t*): maternal BMI (kg/m^2^) = BMI − 25.04701852; TNF-α (pg/mL) = TNF-α − 6.897036576; hepcidin (µg/L) = ln(*X*) + 0.507286449, where *X* = hepcidin/10. Model VIF: 1.05; condition number: 3.14, *n* = 134.

3 Model B: Variables that entered ≥500 bootstrap repetitions but were taken out by the MFP process: maternal age (y), hematocrit (%), IL-10 (pg/mL), TNF-α (pg/mL), vitamin D (nmol/L), serum iron (µg/L). The MFP process provided the following equations for transforming covariates (*t*): pulse pressure (mm Hg) = pulse pressure − 40.71264368; lymphocyte count (×10^3^/mm^3^) = lymphocyte count − 1.992758632; IL-17 (pg/mL) = IL-17 – 6.348457459; MCP-1 (pg/mL) = MCP-1 – 200.2413243. Model VIF: 1.07; condition number: 3.62, *n* = 87.

4 Model C: Variables that entered ≥500 repetitions but were taken out by the MFP process: diastolic blood pressure (mm Hg), RBC count, serum iron (µmol/L), plasma volume (L). The MFP process provided the following equations for transforming covariates (*t*): maternal age (y) = maternal age − 24.95505618; fieldwork (h/d) = fieldwork − 2.04494382; ferritin (mg/L) = ferritin − 13.44617976; IL-12 (pg/mL) = IL-12 – 16.30536148. Model VIF = 1.05; condition number = 2.97, *n* = 89. VIF: variance inflation factor as measurement of collinearity, values <10 were accepted. Condition numbers <30 were accepted as measurement of stability of coefficients.

**FIGURE 3:**
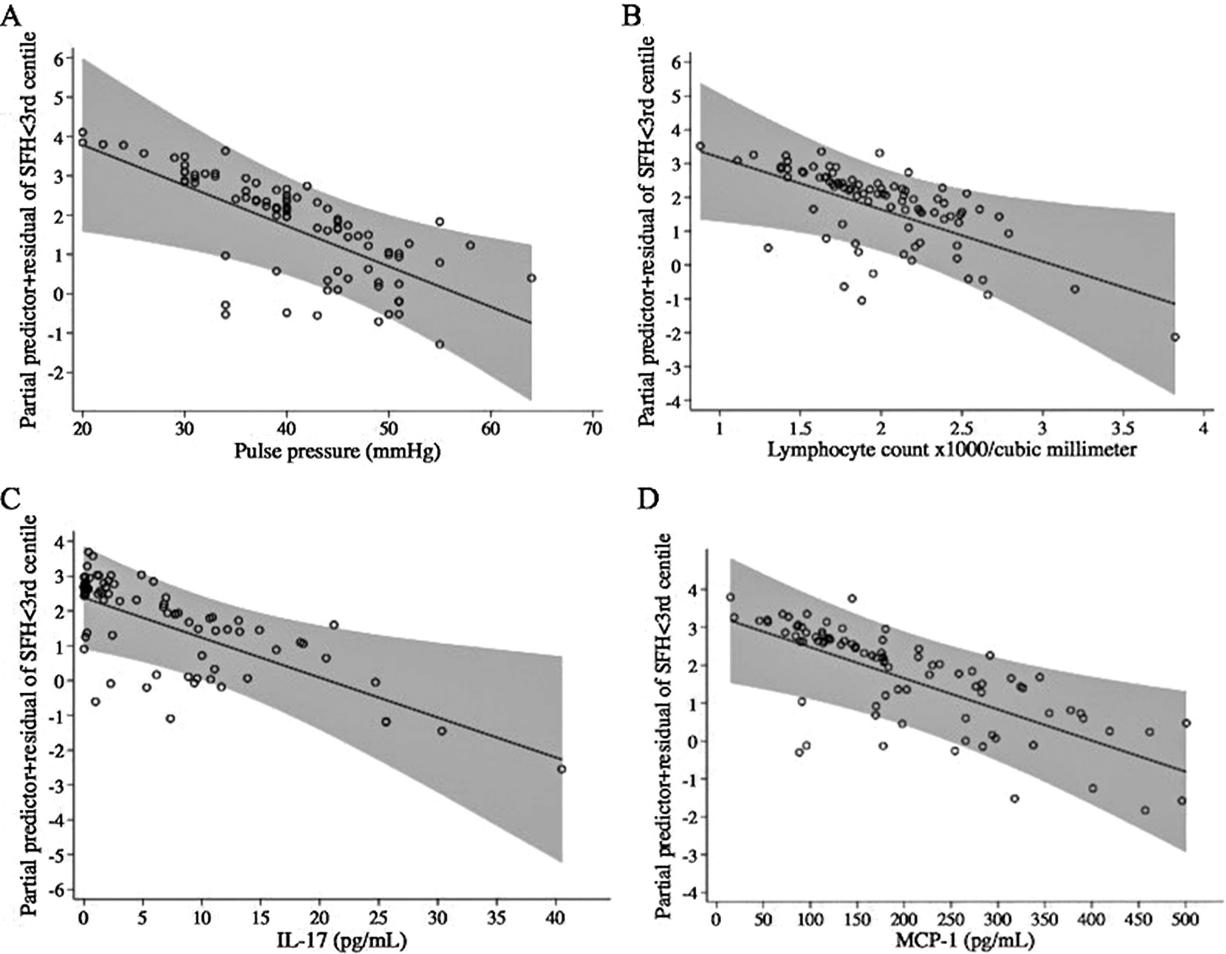
Fractional polynomial logistic regression lines with 95% CIs (gray areas) for associations of pulse pressure (mm Hg) (A), lymphocyte count (B), IL-17 (pg/mL) (C), and MCP-1 (pg/mL) (D) with SFH <3rd centile compared with SFH 3–10 using INTERGROWTH-21 standards, adjusted for all covariates in the model. MCP-1, monocyte chemotactic protein 1; SFH, symphysis–fundal height.

In a third model, we compared an SFH <3rd centile with SFH 3–10 (Table 4B). Higher pulse pressure and an increased inflammatory response indicated by higher lymphocyte count and higher concentrations of IL-17 and the chemokine MCP-1 were associated with decreased odds of an SFH <3rd centile when compared with SFH 3–10. Regression lines of associations between an SFH <3rd centile with pulse pressure, lymphocytes, IL-17, and MCP-1 are shown in **Figure 3**.

**FIGURE 4:**
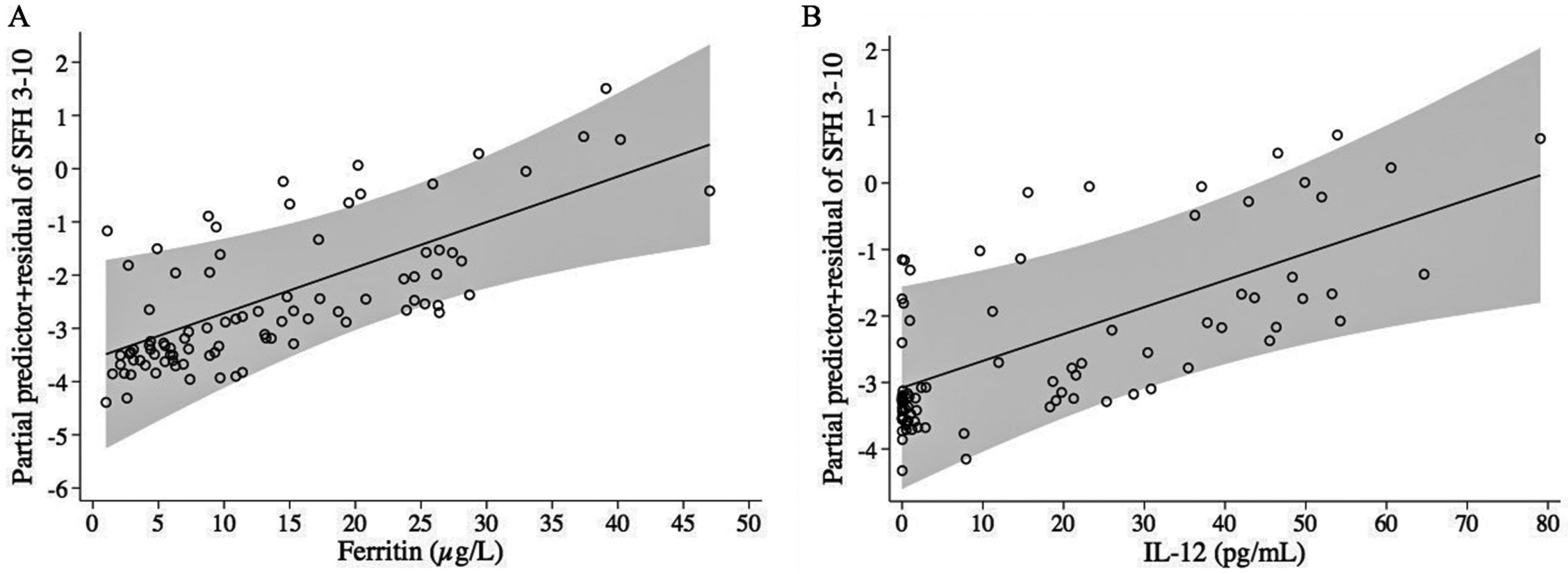
Fractional polynomial logistic regression lines with 95% CIs (gray areas) for associations of ferritin (µg/L) (A) and IL-12 (pg/mL) (B) with SFH 3–10 compared with SFH 10–90 using INTERGROWTH-21 standards, adjusted for all covariates in the model. SFH 3–10, symphysis–fundal height ≥3rd to <10th centiles; SFH, 10–90, symphysis–fundal height ≥10th to ≤90th centiles.

The MFP model comparing SFH 3–10 with normal SFH (10–90) is shown in Table 4C. Older maternal age in years was associated with decreased odds of SFH 3–10, whereas higher IL-12 and higher ferritin were associated with increased odds of SFH 3–10. In **Figure 4**, postestimation regression lines showing the odds of SFH 3–10 with higher ferritin and IL-12 are presented.

### Model for CLAP and INTERGROWTH SFH >90th centile

By using the MFP algorithm, no variables emerged as being significantly associated with SFH >90th centile using either CLAP or INTERGROWTH standards (models not shown).

### MFP linear regression models for hepcidin

Given the unexpected association found between higher hepcidin with an SFH <3rd centile, we explored associations of hepcidin with MINDI variables while controlling for GA (weeks) and SFH (centimeters) in a model shown in **Table 5**. We observed that hepcidin was inversely associated with SFH in centimeters, after adjusting for other variables, including GA. Moreover, nutrient indicators (higher intake of MNS, higher ferritin), a lower urinary pH, and inflammation (higher CRP and IL-17) were associated with higher hepcidin and explained 40.1% of its variability. No infections entered our bootstrap procedure for inclusion into MFP models.

**TABLE 5:** MFP linear regression model for hepcidin^1^

Log hepcidin	Coefficient ± SE	*P*	95% CI	β	Overall model
*t* GA, wk	0.01 ± 0.003	0.010	0.002, 0.01	0.28	*P* < 0.0001, adjusted *R*^2 ^= 0.401
*t* SFH, cm	–0.03 ± 0.01	0.004	–0.05, –0.01	–0.32	
*t* MNS intake (tbsp/d)	0.05 ± 0.02	0.035	0.004, 0.10	0.14	
*t* MAP, mmHg	0.001 ± 0.005	0.783	–0.01, 0.01	0.02	
*t* CRP, mg/L	0.03 ± 0.01	<0.0001	0.01, 0.05	0.23	
*t* IL-17, pg/mL	0.01 ± 0.005	0.012	0.003, 0.02	0.16	
*t* Urinary pH	–0.12 ± 0.04	0.008	–0.21, –0.03	–0.17	
*t* Ferritin, µg/L	2.14 ± 0.05	<0.0001	2.05, 2.23	0.40	
Constant	2.14 ± 0.05	<0.0001	2.05, 2.23		

1 Variables that entered ≥500 repetitions but were taken out by the MFP process: parity, coffee intake (cups/d), MAP (mm Hg), RBC count, number of monocytes and eosinophils, TNF-α (pg/mL), presence of scabies, scores of *Lactobacillus*, and vitamin B-12 (pmol/L). The MFP process provided the following equations for transforming covariates (*t*): *t* GA (wk) = *X*^3^ – 26.13282964, where *X* = GA/10; *t* SFH (cm) = SFH − 26.86060606; *t* MNS (tbsp/d) = MNS − 1.375757576; *t* MAP (mm Hg) = MAP − 75.11313162; *t* CRP (mg/L) = CRP − 4.92727271; *t* IL-17 (pg/mL) = IL-17 – 6.413284447; *t* Urinary pH = pH − 6.415151515; *t* Ferritin (µg/L) = ln(*X*) + 1.833487176, where *X* = ferritin/100. Model VIF = 1.65; condition number = 3.74, *n* = 165. VIF: variance inflation factor as measurement of collinearity, values <10 were accepted. Condition numbers <30 were accepted as measurement of stability of coefficients. CRP, C-reactive protein; GA, gestational age; MAP, mean arterial pressure; MFP, multivariable fractional polynomial; MNS, multiple nutrient supplement; SFH, symphysis–fundal height; tbsp, tablespoons.

## Discussion

SFH is widely used in developing countries to screen for SGA with a reported specificity of ≥80% (1, 58, 59). However, the utility of SFH goes beyond the prediction of SGA, given its association with not only fetal weight but also amniotic fluid volume and GA (3, 19). Our goal had been to apply both CLAP and the new international INTERGROWTH standards for SFH (12) to our MINDI cohort. Based on CLAP standards, low SFH was detected in only 8% of our study population, whereas >50% of our study population had an SFH <10th centile based on the more recent international INTERGROWTH standards. A recent study assessing the prevalence of SGA (birthweight <10th centile) using the INTERGROWTH standards in low- and middle-income countries reported the highest world estimates of 34% for South Asia (60). In contrast, in our study, the prevalence of an SFH <3rd centile was higher and approached 40% in the Ngäbe-Buglé indigenous community in Panama. Previously, maternal infection/inflammation (61–63) and decreased plasma volume expansion or hypovolemia (38, 64) had been associated with both SGA infants and low amniotic fluid volume. Maternal malnutrition also had been associated with SGA (65). In the present study we report associations of maternal infections, inflammation, and nutrient deficiencies with SFH, which highlights the potential of SFH to assist in the identification of factors contributing to the etiology of high-risk pregnancies in resource-poor settings.

In our MINDI cohort, 4 novel findings emerged:

1. Hepcidin was the main determinant of an SFH <10th centile (CLAP) and an SFH <3rd centile (INTERGROWTH), and hepcidin was associated with indicators of inflammation (ferritin, IL-17, CRP) and indirect indicators of hypovolemia and malnutrition (lower urinary pH and higher intake of MNS).
2. In contrast, higher pulse pressure and TNF-α were associated with lower odds of an SFH <3rd centile (INTERGROWTH) and higher TNF-α also decreased the odds of an SFH <10th centile (CLAP).
3. Both wood smoke and *Trichuris* infection were associated with low SFH.
4. Inflammation biomarkers were associated with SFH; higher ferritin and IL-12 increased the odds of SFH 3–10 compared with SFH 10–90, whereas higher lymphocyte counts, higher IL-17 and higher MCP-1 were associated with decreased odds of SFH <3rd centile when compared with SFH 3-10.

**FIGURE 5:**
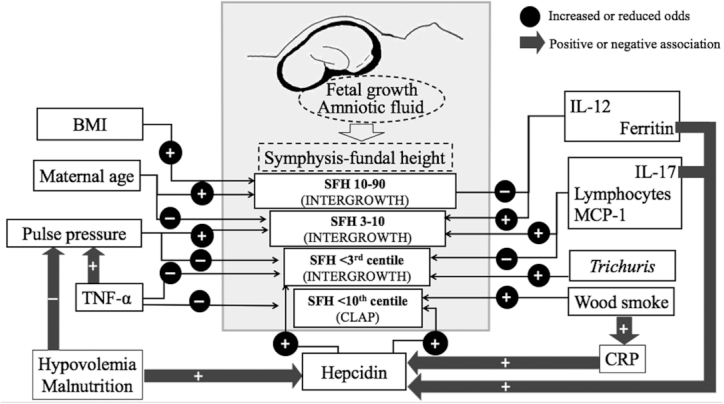
Significant associations of SFH, reflecting fetal growth and amniotic fluid volume, with MINDI. Gray arrows with positive (+) and negative (–) signs indicate positive and negative associations, respectively. Associations of pulse pressure with TNF-α (25) and of CRP with wood smoke (24) had been reported previously. Black arrows with positive and negative signs inside black circles indicate factors that increased or decreased the odds of SFH between the 10th and the 90th centiles (SFH 10–90), between the 3rd and 10th centiles (SFH 30–10), and of <3rd centile using the INTERGROWTH-21 standards, and the odds of SFH <10th centile using the CLAP standards. CLAP, Latin American Center of Perinatology; CRP, C-reactive protein; MINDI, maternal infections, nutrient deficiencies, and inflammation; SFH, symphysis–fundal height.

These findings are highlighted in **Figure 5**.

### Determinants of hepcidin provide insight to its association with SFH

Hepcidin, a protein synthetized in the liver, is the main modulator of iron concentrations in response to elevated iron (66), and under inflammatory conditions an increase in hepcidin inhibits intestinal iron absorption and the release of iron from recycling macrophages (67). Hepcidin is suppressed in a healthy pregnancy (68–71), which favors the supply of iron for augmented erythropoiesis and for fetal growth (72). In our population, hepcidin was not suppressed, was higher in women with lower SFH, and emerged as the main determinant of an SFH <10th centile using CLAP and an SFH <3rd centile using INTERGROWTH. Furthermore, our MFP regression model for hepcidin showed independent associations of hepcidin with ferritin, CRP, IL-17, urinary pH, and the intake of MNS, aligning with previous reports in the literature where hepcidin was associated with ferritin in pregnant Gambian women (73); however, evidence of the association of hepcidin with infection, inflammation, and malnutrition during pregnancy or with SFH has not been described.

Not unexpectedly, higher hepcidin was associated with higher ferritin, as previously described (74). However, in the present study, the majority of these pregnant indigenous women had ferritin <20 µg/L, an indicator of depleted iron stores (40), making it unlikely that hepcidin in these pregnant women was responding to elevated iron concentrations (75). We show that other factors, including inflammation, hypovolemia, and malnutrition, were associated with higher hepcidin in this marginalized community where co-existing infections, nutrient deficiencies, and inflammation were common.

#### Inflammation

No association between hepcidin and CRP has been reported in healthy Eurasian (76), European (69), or African (73) pregnant women. However, during inflammation, hepcidin can increase in pregnant women even if iron deficiency is present (77). In our study, CRP was positively associated with hepcidin; thus, the higher hepcidin may reflect its association with this biomarker of inflammation. Moreover, among the cytokines we measured, IL-17 was the only one entering the hepcidin model. Although both IL-17 and hepcidin increase during infections (78), the association of hepcidin with IL-17 in our poly-infected pregnant women is novel and requires further investigation.

#### Urinary pH

In this population of pregnant mothers with low plasma volumes we found a consistent association of hepcidin with lower urinary pH, another indicator of volemic status (51), suggesting that hemoconcentration, previously reported in this population (25), may contribute to higher hepcidin concentrations.

Urinary pH also reflects the serum acid–base state (49). Based on experimental studies, it has been proposed that metabolic acidosis may contribute to the elevation of hepcidin in clinical conditions (79), and it is known that fasting-induced metabolic acidosis with ketone body production is more frequent in the third trimester of pregnancy due to increased energy requirements (80). An acidic environment occurs in the presence of fasting (81), and therefore may be another condition that contributes to our observation of higher hepcidin in our population. In support of hepcidin being higher due to fasting, we observed an association of higher hepcidin with higher MNS intake, which may reflect an energy deficit. During our visits to the field, we noticed that mothers largely relied on MNS, sometimes as the sole source of nutrition. Our findings also provide evidence that nutritional supplementation programs in vulnerable populations are modest at best (82).

Collectively, these results confirm associations of hepcidin with several biomarkers of inflammation, hypovolemia, and nutritional status and highlight the possibility that hepcidin may be a potential marker of maternal–fetal well-being when infections, inflammation, and malnutrition co-exist.

### Other predictors of low SFH

A second novel factor that emerged in our study was the association of decreased odds of both an SFH <10th centile from CLAP and an SFH <3rd centile from INTERGROWTH with higher TNF-α. We had previously reported that higher pulse pressure was associated with higher TNF-α and that pulse pressure <30 mm Hg, an indicator of low peripheral perfusion, was associated with lower SFH *z* scores (25). Although TNF-α concentrations may also reflect fetal antimicrobial defense against infection (83), TNF-α is known to increase during normal pregnancy (84) and has also been recognized as a critical factor for placental development (85). TNF-α has been shown to be high in SGA associated with preeclampsia, but not with idiopathic SGA (86), and in intrauterine growth retardation (IUGR) associated with placental insufficiency but not in those with IUGR with normal placental perfusion (87). Given that the increase in TNF-α in pregnancies complicated with preeclampsia is probably a consequence of placental hypoxia (88), our results support the possible role of this cytokine in the compensatory increase in uterine perfusion under low plasma volume and low pulse pressure conditions as an explanation of its association with decreased odds of an SFH <3rd centile.

### Nutrition and SFH

SFH has been previously reported as an indicator of maternal nutritional status. In a Malawian study of moderate-malnutrition pregnancies, SFH was lower in adolescent compared with adult females (89). Similarly, we found that an older maternal age decreased the odds of SFH 3–10 and that higher BMI decreased the odds of an SFH <3rd centile. In our study population, diet questionnaires showed the low intake of animal foods, the main source of vitamin B-12 (90), which, together with low concentrations of the vitamin and of RBP, an indicator of protein status (47), strongly support our observation of protein malnutrition in these indigenous mothers.

Even though the need for iron to promote fetal and future infant development is well known (91), the effect of iron deficiency on fetal growth is somewhat controversial. An older meta-analysis on the effect of iron supplementation during pregnancy commissioned by the WHO found that iron supplementation increased birth weight by 100 g at most (92). This meta-analysis also acknowledged limitations, as only 1 study provided solid evidence of a positive effect of iron supplementation on birth weight and that both high and low hemoglobin concentrations were associated with smaller infants (92). However, given that maternal iron deficiency is often associated with impaired fetal growth and development (93), we might have expected to find an association of higher SFH with iron supplementation, or an association of an SFH <10th centile with lower iron status. However, iron supplementation did not enter our models for low SFH, whereas higher ferritin increased the odds of SFH 3–10 compared with SFH 10–90. It is likely that ferritin in our population is influenced by inflammation and may not only reflect maternal iron status.

There is evidence that elevated ferritin is associated with impaired fetal growth. Ferritin concentrations >48 µg/L have been proposed as a predictor of IUGR, whereas concentrations <22 µg/L would be indicative of constitutional SGA fetuses (94), an approach that was not possible to apply in our study because most women were iron deficient and only 8% reached ferritin values >48 µg/L. Our findings do support current Panamanian guidelines for iron supplementation during pregnancy, which preclude the use of iron in women with infections (95) due to the risk of exacerbation of some infections (96).

### SFH 3–10 contrasts with SFH ≤3rd centile

We were intrigued by the observation that an SFH <3rd centile and SFH 3–10 had different determinants when compared with SFH 10–90. Whereas the odds of an SFH <3rd centile were increased by hepcidin and decreased by higher BMI and TNF-α, higher ferritin and IL-12 distinguished SFH 3–10 from normal SFH 10–90. Moreover, a higher pulse pressure and inflammation indicators (higher lymphocytes, IL-17, and MCP-1) distinguished SFH 3–10 from SFH <3rd centile, suggesting that intrauterine perfusion and/or inflammation underscored differences and a possible different etiology of impaired intrauterine growth between these 2 groups.

In our model for SFH 3–10 compared with normal SFH (10–90), higher ferritin and IL-12 emerged as increasing the odds of SFH 3–10. It has been reported that the absence of a decline in ferritin concentrations as pregnancy progresses is associated with increased odds of chorioamnionitis (97). Also, IL-12 has been proposed as a biomarker of chorioamnionitis caused by *Ureaplasma urealiticum* (98), a common cause of urogenital infection (99). Furthermore, the possibility of subclinical chorioamnionitis is supported by our finding that cytokines increased the odds of SFH 3–10 when compared with an SFH <3rd centile. Three other biomarkers entering this model—lymphocyte counts (100, 101), IL-17 (102), and MCP-1 (103)—have been all linked with intra-amniotic infection. This subclinical condition may precede chorioamnionitis, which usually develops from urogenital infections, particularly bacteria associated with urinary infection (104) and bacterial vaginosis (105), which were present in a large majority (64% and 61%, respectively) in our study population.

The association of higher ferritin increasing the odds of SFH 3–10 compared with SFH 10–90 continues to support the inflammatory state associated with SFH 3–10. Higher ferritin has been suggested as a marker of infection in women with preterm rupture of membranes at <32 weeks of gestation (106). Moreover, maternal iron status indicated by higher ferritin at the beginning of pregnancy has been associated with better pregnancy outcomes, including higher birth weight; however, higher ferritin at the end of pregnancy has been associated with smaller birth size in developing countries (75). Others have associated higher periconceptional ferritin with preterm birth in women from Canada (107). Further exploration of ferritin as a biomarker of impaired fetal growth in marginalized communities is warranted based on our findings, with accompanying assessment of both iron and inflammatory parameters.

We also found it intriguing that in these women who were experiencing multiple bacterial, protozoan, and nematode infections, a lower protein status may have contributed to more women having an SFH <3rd centile as compared with those with SFH 3–10. Protein deficiency is known to impair the inflammatory response (108). There is experimental evidence of decreased IL-4 under maternal protein deprivation and during intestinal nematode infections (109). Others have reported that both T-helper (Th) 1 and Th-2 cytokines are negatively correlated with BMI in malnourished, noninfected, nonpregnant individuals (110) and it has also been described that Th-1 cytokines including IL-12 are lower in malnourished children (111). We suggest that the higher prevalence of protein deficiency in the <3rd-centile group may modulate immune responses, as SFH centile for GA was also lower in these protein-deficient women. In our study with poly-infected pregnant women, we also found lower eosinophils, basophils, and IL-4 but higher IL-10 and IL-13 in protein-deficient women, highlighting the complex interaction of protein malnutrition and immunity during pregnancy under MINDI conditions.

### Other factors associated with low SFH

#### Infections

Among the range of infections present in our population, only the presence of *Trichuris* was associated with increased odds of an SFH <3rd centile. Of note, in all women with *Trichuris* infection, hookworm coinfection was present. A possible explanation for the association of these nematodes with an SFH <3rd centile could be blood loss due to both hookworm (112) and *Trichuris* infection (113) leading to anemia. However, none of the RBC indices were associated with low SFH. Chronic *Trichuris* infections can experimentally elicit a persistent Th-1 immune response (114), suggesting that the associations of *Trichuris* with an SFH <3rd centile may be linked to inflammation.

#### Wood smoke

In Guatemala, it was found that women cooking with open fires had infants with lower birth weights than those cooking with chimney stoves or those cooking with electricity or gas (115). Increased environmental carbon monoxide leading to the formation of carboxyhemoglobin is considered to be the common mechanism by which cigarette smoking contributes to low birth weights (115). Consistent with this, our study found that more hours of exposure per day to wood smoke was associated with the lowest SFHs detected by the CLAP <10th centile. Moreover, we had previously shown that wood smoke was associated with elevated CRP in this population (24). Given the known association of smoking with low birth weight (116), and the specificity of the CLAP standards for the detection of SGA, our findings highlight the need to target wood-smoke exposure as part of public health policies to decrease inflammation and improve pregnancy outcomes.

### Conclusions

This study identified, for the first time, determinants of low SFH in the context of multiple infections, nutrient deficiencies, and inflammation in a marginalized community of pregnant indigenous women. Of interest, our study shows that hepcidin is associated with low SFH in the presence of malnutrition, inflammation, and with a nematode infection and also that INTERGROWTH standards provided the rare opportunity to study associations of MINDI with women having all ranges of SFH. We provide evidence that a low SFH in a MINDI context should alert clinicians to the possibility of inadequate plasma volume expansion, subclinical inflammation, and maternal malnutrition. Public health interventions leading to dietary improvements and increasing our understanding of the complexity of the inflammatory response associated with multiple infections may be important if we are to improve maternal and fetal health. The use of low SFH as a screening tool in remote settings for the identification of high-risk pregnancies is a first step.

## Supplementary Material

nzab012_Supplemental_File

## Notes

Supported by the “Secretaría Nacional de Ciencia, Tecnología e Innovación” (SENACYT) Panama (COL08-009) (EM, MES, KGK), the McGill Vitamin Fund (MES, KGK), and the NIH (EM, KGK).

Author disclosures: The authors report no conflicts of interest.

Supplemental Tables 1–3 are available from the “Supplementary data” link in the online posting of the article and from the same link in the online table of contents at https://academic.oup.com/cdn/.

Abbreviations used: CLAP/WR, Latin American Center of Perinatology, Women and Reproductive Health; CRP, C-reactive protein; DBP, diastolic blood pressure; GA, gestational age; IUGR, intrauterine growth retardation; MAP, mean arterial pressure; MCP-1, monocyte chemotactic protein 1; MFP, multivariable fractional polynomial; MINDI, multiple infections, nutrient deficiencies, and inflammation; MNS, multiple nutrient supplement; PAHO, Pan-American Health Organization; RBP, retinol-binding protein; SBP, systolic blood pressure; SFH, symphysis–fundal height; SGA, small-for-gestational-age; sTfR, serum transferrin receptor; tbsp, tablespoons; TBV, total blood volume; Th, T-helper; WBC, white blood cell.
