# Supplementary material for: INTERGROWTH-21 Identifies High Prevalence of Low Symphysis–Fundal Height in Indigenous Pregnant Women Experiencing Multiple Infections, Nutrient Deficiencies, and Inflammation: The Maternal Infections, Nutrient Deficiencies, and Inflammation (MINDI) Cohort
Source: Curr Dev Nutr. 2021 Apr 12;5(4):nzab012. doi: 10.1093/cdn/nzab012 (PMC8053398; doi:10.1093/cdn/nzab012)
Supplement: nzab012_Supplemental_File [file nzab012_supplemental_file.doc]

**INTERGROWTH-21 identifies high prevalence of low symphysis-fundal height in indigenous pregnant women experiencing multiple infections, nutrient deficiencies and inflammation: the MINDI cohort. González-Fernández et al.**

**Supplementary material**

**Supplemental Table S1** Comparison of maternal characteristics by symphysis-fundal height (SFH) classification according to centiles for gestational age using CLAP/WR standards.

| **SFH according to CLAP/WR,**  **n (%)** | **<10^th^ centile**  **14 (8%)** | **≥10^th^ – ≤90^th^**  **110 (63.2%)** | **>90^th^ centile**  **50 (28.7%)** | **p-value** |
| --- | --- | --- | --- | --- |
| BMI | 25.5 (21.1 – 27.5) | 24.3 (22.3 – 26.8) | 25.1 (23.4 – 27.2) | 0.57 |
| Underweight | 5(e2) | 16 (e13) | 0 (e6) | <0.0001 |
| Normal weight | 5 (e9) | 75 (e70) | 31 (e32) | 0.063 |
| Overweight | 4 (e3) | 19 (e27) | 19 (e12) | 0.015 |
| Gestational age | 30 (18 – 39) | 30 (23 – 36) | 32 (26 – 39) | 0.114 |
| Plasma volume, mL^(3)^ | 2138 (1996 – 2291) | 2091 (1979 – 2239) | 2132 (2027 -2235) | 0.537 |
| Urinary pH (mean ± SD) | 5.8 ± 0.7^b^ | 6.5 ± 0.9^a^ | 6.4 ± 0.9^ab^ | 0.015 |
| Urinary specific gravity ≥1020 | 4 (e4) | 26 (e27) | 13 (e12) | 0.888 |
| Wood smoke exposure | 14 | 104 | 43 | 0.150 |
| **Blood pressure (mmHg)** |  |  |  |  |
| Systolic | 100 (91 – 105) | 101 (96 – 110) | 103 (99 – 111) | 0.434 |
| Diastolic | 63 (58 – 68) | 60 (55 – 67) | 61 (58 – 69) | 0.617 |
| MAP | 75 (72 – 80) | 73 (69 – 81) | 77 (71 – 83) | 0.580 |
| Pulse pressure | 37 (30 – 46) | 40 (34 – 47) | 40 (35 – 47) | 0.363 |
| **Inflammation indicators** |  |  |  |  |
| CRP (mg/L) | 3.1 (1.1 – 4.3) | 3.2 (1.5 – 6.4) | 4.3 (1.6 – 6.7) | 0.532 |
| Cytokines (pg/L) (n=173) |  |  |  |  |
| IL-1β | 0.5 (0.1 – 1.3)^b^ | 1.9 (0.5 – 6.9)^ab^ | 2.8 (0.6 – 9.8)^a^ | 0.014 |
| IL-4 | 3.2 (2.1 – 10.2) | 5.5 (3.2 – 22.7) | 5.0 (0.7 – 18.8) | 0.308 |
| IL-6 | 1.6 (0.5 – 6.8) | 1.6 (1.0 – 13.7) | 2.9 (1.6 – 13.1) | 0.246 |
| IL-10 | 1.3 (0.4 – 1.6) | 1.6 (0.1 – 5.1) | 1.1 (0.1 – 4.4) | 0.660 |
| IL-12 | 0.3 (0.02 – 5.9) | 1.9 (0.02 – 26.3) | 2.1 (0.1 – 28.7) | 0.300 |
| IL-13 | 1.6 (0.1 – 1.6) | 1.6 (0.1 – 8.5) | 1.6 (0.1 – 6.8) | 0.663 |
| IL-17 | 1.7 (0.1 – 7.7) | 2.1 (0.1 – 10.9) | 2.3 (0.1 – 11.2) | 0.872 |
| IFN-γ | 1.4 (0.8 – 5.7) | 3.8 (1.0 – 13.9) | 5.0 (1.0 – 13.6) | 0.322 |
| TNF-α | 0.9 (0 – 1.3)^b^ | 7.1 (0.02 – 12.4)^a^ | 8.2 (0.04 – 14.2)^a^ | 0.007 |
| MCP-1 | 134.6 (105.9 – 177.4) | 170.9 (112.6 – 258.4) | 192.2 (124.4 – 300.9) | 0.149 |
| **Iron status indicators** |  |  |  |  |
| Ferritin, µg/L | 19.0 (6.3 – 55.3) | 8.9 (5.5 – 19.5) | 12.2 (6.1 – 19.7) | 0.127 |
| Ferritin <20 µg/L | 7 (e10) | 83 (e81) | 38 (e37) | 0.113 |
| Serum iron, µmol/L | 11.7 (5.4 – 15.9) | 8.4 (5.4 – 12.0) | 8.2 (6.0 – 13.5) | 0.826 |
| Serum iron <8.9 µmol/L | 6 (e7) | 59 (e59) | 28 (e27) | 0.683 |
| sTfR, mg/L | 4.5 (3.3 – 6.5) | 5.2 (4.0 – 7.4) | 5.5 (4.0 – 8.4) | 0.282 |
| sTfR >8.3 mg/L | 1 (e3) | 18 (e20) | 13 (e9) | 0.232 |
| Hepcidin, µg/L | 14.2 (6.9 – 24.4)^a^ | 7.6 (4.9 – 11.0)^b^ | 6.8 (5.0 – 9.4)^b^ | 0.044 |
| **Nutrients** |  |  |  |  |
| Folic acid, nmol/L | 10.4 (7.5 – 18.1) | 13.7 (9.9 - 20.7) | 13.6 (10.3 – 16.6) | 0.247 |
| Folic acid <10 nmol/L | 6 (e4) | 28 (e29) | 12 (e13) | 0.342 |
| Vitamin B_12_, pmol/L | 100.5 (82 – 117) | 97.5 (78 – 126) | 92 (79 – 108) | 0.275 |
| Vitamin B_12_ <150 pmol/L | 14 (e12) | 95 (e98) | 46 (e44) | 0.266 |
| Vitamin D, nmol/L | 51.9 (39.0 – 36.4) | 42.9 (34.6 – 56.7) | 42.8 (30.2 – 51.1) | 0.139 |
| Vitamin D <50 nmol/L | 4 (e8) | 69 (e69) | 36 (e31) | 0.013 |
| Vitamin A, µmol/L (n= 172) | 1.1 (1.0 – 1.4) | 1.2 (0.9 – 1.3) | 1.1 (0.9 – 1.4) | 0.788 |
| Vitamin A <1.05 µmol/L (n= 172) | 4 (e6) | 44 (e45) | 24 (e21) | 0.424 |
| RBP, mg/L (n= 173) | 3.3 (2.7 – 3.9) | 4.8 (2.7 – 8.6) | 4.4 (2.9 – 8.5) | 0.159 |
| RBP <30 mg/L (n= 173) | 5 (e4) | 32 (e31) | 13 (e14) | 0.766 |

^(1)^ Abbreviations: BMI, body mass index; CRP, C-reactive protein; RBP, retinol-binding protein

^(2)^ One-way ANOVA was used to compare normally-distributed variables, Kruskal-Wallis test to compare non-normally distributed variables, and Chi^2^ or Fisher’s exact tests to compare proportions; expected frequencies are shown in parenthesis (e#). Longitudinal variables are presented as median (IQR). Binary variables are reported as frequencies (expected frequency). Superscripts “a” and “b” denote significant post-hoc differences among groups (p<0.05).

^(3)^ Plasma volume was calculated as total blood volume (TBV) * (1 – hematocrit) (119). TBV was calculated using Nadler’s equation (TBV=0.3561 x (Ht in M)^3 + 0.03308 x Wt in kgs +0.1833) (120).

**Supplemental Table S2** Comparison of maternal characteristics by symphysis-fundal height (SFH) classification according to centiles for gestational age.

| **SFH according to INTERGROWTH-21**  **n (%)** | **<3^rd^**  **66 (37.9%)** | **≥3^rd^ – <10^th^**  **22 (12.6%)** | **≥10^th^ – ≤90^th^**  **70 (40.2%)** | **>90^th^**  **16 (9.2%)** | **p** |
| --- | --- | --- | --- | --- | --- |
| BMI | 23.8 (21.4–26.1)^b^ | 25.2 (22.9–26.4)^ab^ | 26.0 (23.4–28.5)^a^ | 24.5 (21.9–26.9)^ab^ | 0.006 |
| Underweight | 13 (e8) | 2 (e3) | 6 (e8) | 0 (e2) | 0.095 |
| Normal weight | 39 (e42) | 17 (e14) | 47 (e45) | 8 (e10) | 0.259 |
| Overweight | 14 (e16) | 3 (e5) | 17 (e17) | 8 (e4) | 0.080 |
| Gestational age | 33 (22.5–38.4)^a^ | 35.1 (28.1–37.6)^a^ | 30.2 (23.3–35.1)^ab^ | 24.7 (20.6–27.6)^b^ | 0.007 |
| Plasma volume, mL^1^ | 2067  (1987 – 2215) | 2086  (1979 – 2172) | 2136  (2008 – 2260) | 2163  (2061 – 2219) | 0.219 |
| Urinary pH (mean ± SD) | 6.2 ± 0.8^b^ | 6.5 ± 0.9^ab^ | 6.5 ± 0.9^ab^ | 6.8 ± 1.0^a^ | 0.040 |
| Urinary specific gravity ≥1020 | 19 (e16) | 4 (e6) | 16 (e18) | 4 (e4) | 0.654 |
| Wood smoke exposure | 62 (e61) | 21 (e20) | 66 (e65) | 12 (e15) | 0.048 |
| Blood pressure (mmHg) |  |  |  |  |  |
| Systolic | 100 (94 – 110) | 105 (101 – 112) | 103 (97 – 110) | 100 (93 – 109) | 0.358 |
| Diastolic | 60 (55 – 68) | 59 (56 – 66) | 61 (55 – 68) | 59 (56 – 64) | 0.838 |
| MAP | 73 (68 – 81) | 73 (70 – 79) | 75 (70 – 82) | 73 (68 – 82) | 0.907 |
| Pulse pressure | 40 (33 – 45) | 45 (40 – 50) | 41 (35 – 47) | 41 (36 – 48) | 0.052 |
| **Inflammation indicators** |  |  |  |  |  |
| CRP (mg/L) | 3.6 (1.7–7.0) | 2.7 (1.3–5.8) | 3.5 (1.4–6.7) | 4.4 (2.2–7.0) | 0.790 |
| Cytokines (pg/L) (n=173) | 66 | 22 | 69 | 16 |  |
| IL1β | 1.1 (0.3–5.6) | 5.8 (1.3–5.7) | 2.3 (0.4–8.5) | 1.3 (0.5–11.9) | 0.095 |
| IL4 | 3.2 (2.3–10.5)^b^ | 11.6 (0.7–34.7)^ab^ | 3.5 (0.7–20.5)^ab^ | 17.6 (9.3–23.4)^a^ | 0.045 |
| IL6 | 1.6 (0.5–6.8)^b^ | 6.5 (1.6–24.0)^a^ | 2.5 (1.6–11.5)^b^ | 7.3 (1.6–25.8)^ab^ | 0.025 |
| IL10 | 1.6 (0.1–1.6) | 2.3 (0.1–6.9) | 1.6 (0.1–5.4) | 0.1 (0.1–6.3) | 0.616 |
| IL12 | 0.7 (0.0–14.7)^b^ | 19.4 (0.9–49.9)^a^ | 1.7 (0.1–25.3)^ab^ | 3.5 (0.0–21.2)^ab^ | 0.018 |
| IL13 | 1.6 (0.1–1.6) | 4.6 (0.1–18.3) | 1.6 (0.1–8.5) | 1.6 (0.1–6.4) | 0.499 |
| IL17 | 1.6 (0.1–7.8)^b^ | 9.5 (2.2–13.9)^a^ | 1.6 (0.1–12.7)^b^ | 1.2 (0.0–12.1)^b^ | 0.024 |
| IFNγ | 1.7 (1.0–8.9) | 9.3 (1.8–15.5) | 6.4 (1.0–14.4) | 3.2 (0.3–18.1) | 0.127 |
| TNFα | 2.1 (0.0–9.7)^b^ | 11.5 (0.0–17.2)^a^ | 7.8 (0.4–12.7)^ab^ | 7.6 (2.2–13.7)^ab^ | 0.010 |
| MCP-1 | 152.7 (105.9–258.4) | 228.5 (177.6–297.6) | 171.0 (119.4–282.5) | 146.3 (112.1–217.1) | 0.063 |
| **Iron status indicators** |  |  |  |  |  |
| Ferritin, µg/L | 9.4 (5.7–24.7) | 14.7 (6.3–25.9) | 9.5 (4.8–17.3) | 13.0 (7.7–18.8) | 0.383 |
| Ferritin <20 µg/L | 46 (e49) | 14 (e16) | 55 (e51) | 13 (e12) | 0.390 |
| Serum iron, µmol/L | 8.4 (5.4–14.2) | 11.4 (5.4–20.3) | 8.4 (5.5–12.0) | 7.9 (5.7–11.6) | 0.457 |
| Serum iron <8.9 µmol/L | 34 (e35) | 10 (e12) | 38 (e37) | 11 (e9) | 0.534 |
| sTfR, mg/L | 5.3 (3.6–7.2) | 4.9 (4.3–7.2) | 5.7 (4.0–7.9) | 4.5 (3.6–6.5) | 0.545 |
| sTfR >8.3 mg/L | 10 (e12) | 3 (e4) | 16 (e13) | 3 (e3) | 0.661 |
| Hepcidin, µg/L | 8.9 (5.6–13.3)^a^ | 6.7 (5.7–17.2)^ab^ | 6.7 (4.3–9.4)^b^ | 7.1 (6.0–8.7)^ab^ | 0.012 |
| **Nutrients** |  |  |  |  |  |
| Folic acid, nmol/L | 12.4 (9.6–19.4) | 13.5 (10.5–16.4) | 14.6 (10.6–18.5) | 12.2 (8.5–18.2) | 0.644 |
| Folic acid <10 nmol/L | 20 (e17) | 4 (e6) | 16 (e18) | 6 (e4) | 0.441 |
| Vitamin B_12_, pmol/L | 106.0 (87.0–128.0)^a^ | 91.5 (78.0–111.0)^ab^ | 93.5 (75.0–117.0)^b^ | 90.0 (76.5–100.0)^ab^ | 0.042 |
| Vitamin B_12_ <150 pmol/L | 58 (e59) | 20 (e20) | 63 (e62) | 14 (e14) | 0.965 |
| Vitamin D, nmol/L | 48.5 (34.6–57.9) | 36.1 (30.7–48.3) | 42.7 (33.8–54.6) | 45.0 (34.9–51.1) | 0.226 |
| Vitamin D <50 nmol/L | 34 (e41) | 17 (e14) | 47 (e44) | 11 (e10) | 0.095 |
| Vitamin A, µmol/L (n= 172) | 1.1 (0.9–1.4) | 1.1 (1.0–1.3) | 1.2 (0.9-1.3) | 1.2 (0.9-1.5) | 0.996 |
| Vitamin A <1.05 µmol/L (n= 172) | 28 (e27) | 9 (e9) | 28 (e29) | 7 (e7) | 0.990 |
| RBP, mg/L (n= 173) | 35.9 (23.8–71.3)^b^ | 56.4 (34.0–79.0)^ab^ | 43.8 (28.7–77.9)^ab^ | 88.4 (41.3–103.1)^a^ | 0.017 |
| RBP <30 mg/L (n= 173) | 25 (e19) | 4 (e6) | 20 (e20) | 1 (e5) | 0.048 |

^(1)^ Abbreviations: BMI, body mass index; CRP, C-reactive protein; RBP, retinol-binding protein

^(2)^ One-way ANOVA was used to compare normally-distributed variables, Kruskal-Wallis test to compare non-normally distributed variables, and Chi^2^ or Fisher’s exact tests to compare proportions; expected frequencies are shown in parenthesis (e#). Longitudinal variables are presented as median (IQR). Binary variables are reported as frequencies (expected frequency). Superscripts “a” and “b” denote significant post-hoc differences among groups (p<0.05).

^(3)^ Plasma volume was calculated as total blood volume (TBV) * (1 – hematocrit) (119). TBV was calculated using Nadler’s equation (TBV=0.3561 x (Height in Meters)^3 + 0.03308 x Weight in kgs +0.1833) (120).

**Supplemental Table S3 Multivariable fractional polynomial logistic regression model for SFH<3^rd^ centile compared with SFH 10-90 in the subsample of women with intestinal nematode data.**

| 1. **SFH <3^rd^ centile**   **compared with SFH 10-90^1^ including**  **nematodes** | **OR ± SE** | **P** | **95% CI** | **Change in odds for SD increase in X** | **Overall model** |
| --- | --- | --- | --- | --- | --- |
| Trimester (0=2^nd^, 1= 3^rd^ trimester) | 4.85 ± 2.70 | 0.005 | 1.63, 14.43 | 2.17 | p = 0.0003  Pseudo R^2^= 0.171 |
| t Hepcidin, µg/L | 1.11 ± 0.05 | 0.025 | 1.01, 1.21 | 2.00 |
| *Trichuris,* presence (0=no, 1=yes) | 5.52 ± 4.80 | 0.049 | 1.01, 30.29 | 1.78 |
| Constant | 0.31 ± 0.14 | 0.010 | 0.12, 0.75 |  |

^1^ Model VIF: 1.01, condition number: 3.08, n = 78.Variables that entered ≥500 bootstrap repetitions but were taken out by the MFP process: maternal BMI (kg/m^2^), systolic blood pressure (mmHg), TNF-α (pg/mL).

t: Transformations used by the MFP process:

Hepcidin (µg/L) = Hepcidin – 9.60383335

VIF: variance inflation factor as indicator of collinearity. Values <3.0 were accepted.
